# Cardiovascular and Cerebrovascular Complications Following Ponatinib for Philadelphia Chromosome‐Positive Acute Lymphoblastic Leukemia

**DOI:** 10.1155/cric/3296809

**Published:** 2026-09-01

**Authors:** Catherine Boldig, Andrew Herson, Justin Falk, Mitchell Boshkos, David Z. Rose

**Affiliations:** ^1^ Department of Neurology, University of South Florida Morsani College of Medicine, Tampa, Florida, USA, usf.edu; ^2^ Department of Internal Medicine, University of South Florida Morsani College of Medicine, Tampa, Florida, USA, usf.edu; ^3^ Department of Neurology, Emory School of Medicine, Atlanta, Georgia, USA, emory.edu; ^4^ Department of Internal Medicine, Baylor College of Medicine MD Anderson Track, Houston, Texas, USA

**Keywords:** cerebral bypass, coronary bypass, ponatinib, stroke, transient ischemic attack

## Abstract

Ponatinib is a third‐generation BCR:ABL1 tyrosine kinase inhibitor used in selected patients with Philadelphia chromosome‐positive acute lymphoblastic leukemia (Ph+ ALL), particularly those with T315I mutations, or disease resistant, or those intolerant to prior tyrosine kinase inhibitors. Although effective, ponatinib has been associated with arterial occlusive events. We report a 63‐year‐old man with Ph+ ALL who, while receving ponatinib, developed a myocardial infarction requiring quadruple coronary artery bypass grafting, followed by symptomatic intracranial arterial stenosis necessitating superficial temporal artery to middle cerebral artery bypass. Despite multiple cardiovascular risk factors, the chronology of progressive coronary and cerebral vascular disease raised suspicion for ponatinib‐associated vasculopathy, although causality cannot be established. To our knowledge, this is the first reported case of sequential coronary and cerebral surgical revascularization occurring during ponatinib therapy. This case highlights the importance of careful cardiovascular surveillance, multidisciplinary management, and individualized risk‐benefit assessment in patients treated with ponatinib.

**Key Points**

• Ponatinib is a tyrosine kinase inhibitor approved for treatment of Philadelphia chromosome‐positive (Ph+) acute lymphoblastic leukemia (ALL) and CML.
• Ponatinib is associated with a high incidence of vascular side effects, most commonly hypertension.
• Caring for oncologic patients is complex, and a multidisciplinary team is beneficial, as treatment regimens can have adverse effects on other systems in the body.

## 1. Introduction

Ponatinib is a third‐generation tyrosine kinase inhibitor (TKI) used in selected patients with Ph+ ALL, particularly those with T315I‐mutated disease or resistance or intolerance to prior TKIs. Although highly effective, its use is limited by an established association with arterial occlusive events, including cardiovascular, cerebrovascular, and peripheral vascular complications [1]. The mechanism underlying these events remains incompletely understood, and both drug‐related effects and patient‐specific cardiovascular risk factors are thought to contribute.

We report a patient with Ph+ ALL who developed myocardial infarction requiring coronary artery bypass grafting, followed by symptomatic intracranial arterial stenosis requiring superficial temporal artery to middle cerebral artery bypass while receiving ponatinib. Although a causal relationship cannot be absolutely established, the clinical course observed in this patient is consistent with a possible contribution of ponatinib to multifocal arterial disease. This case underscores the need for cardio‐ and neuro‐vascular risk assessment and continued monitoring throughout treatment with ponatinib.

## 2. Case Presentation

A 63‐year‐old man with a medical history of hypertension, hyperlipidemia, diabetes mellitus, prostate cancer, and squamous cell carcinoma of his forehead, and no history of tobacco use, presented in 2016 with fatigue, dyspnea, night sweats, chills, and a perianal infection. His family history is notable for hypertension, diabetes mellitus, and myasthenia gravis in his father and osteoarthritis in his mother. Peripheral blood flow cytometry demonstrated B‐cell ALL with the Philadelphia chromosome t(9;22) (p190 transcript). He was started on steroids and hyper‐CVAD: cyclophosphamide, vincristine, doxorubicin, and dasatinib. He also received intrathecal methotrexate and cytarabine. In July 2016, he received intravenous methotrexate and cytarabine with dasatinib, and again underwent intrathecal methotrexate with cytarabine. CSF flow cytometry was negative. Following two cycles of hyper‐CVAD, he achieved molecular remission and was continued on dasatinib maintenance therapy. Bone marrow biopsy in September 2016 showed relapse. Therefore, in January 2017, he was started on ponatinib 45 mg oral daily and dexamethasone 40 mg on Days 1–4 for 4 weeks, followed by dexamethasone 40 mg weekly. Following initiation of ponatinib therapy, he developed hypertension and was started on lisinopril. He underwent allogenic transplant and started tacrolimus and sirolimus, and blood counts improved. Bone marrow biopsy and flow cytometry in August 2017 were then negative; however, PCR for BCR:ABL1 p190 was positive at 0.0001% concerning for minimal residual disease. In July 2018, he resumed ponatinib 15 mg daily. Over the next few months, PCR levels rose, and ponatinib was increased to 30 mg daily. He followed closely with cardiology during this time requiring four antihypertensive medications. In June 2022, he experienced a myocardial infarction requiring quadruple coronary artery bypass grafting. He resumed ponatinib 15 mg daily, but developed recurrent transient ischemic attacks (TIA) with transient left‐sided weakness, numbness, and dysarthria in February 2023. Each episode lasted about 30 min. Intracranial imaging revealed moderate multifocal arterial narrowing with 80%–85% high‐grade stenosis of the proximal right middle cerebral artery, 75%–80% stenosis of the proximal right anterior cerebral artery, and 65%–70% stenosis of the mid left middle cerebral artery. Ponatinib was discontinued, and aspirin, apixaban, and statin therapies were started. He then underwent right superficial temporal artery to middle cerebral artery bypass with the neurosurgery team in April 2023. Following discontinuation of ponatinib and completion of coronary and cerebral revascularization procedures, no further coronary or cerebrovascular events have been observed during follow‐up.

## 3. Discussion

Ponatinib is a potent third‐generation TKI with activity against the T315I mutation and other resistant BCR:ABL1 variants [2, 3, 4, 5]. Current treatment guidelines recommend ponatinib in selected clinical scenarios, including T315I‐positive disease and patients with resistance or intolerance to prior TKIs, with treatment individualized according to disease characteristics and the anticipated risk of treatment‐related toxicity [2, 3, 4, 5, 6]. The management of Ph+ ALL relies on inhibition of BCR:ABL1 in combination with multiagent chemotherapy and, in appropriate patients, allogenic hematopoietic stem cell transplantation [3, 4]. Throughout treatment, minimal residual disease monitoring remains an essential component of response assessment and therapeutic decision‐making [3].

Despite its clinical efficacy, ponatinib has consistently been associated with an increased risk of arterial occlusive events [1, 2, 4, 6, 7, 8]. The reported incidence of major vascular adverse events reported in the Phase II PACE trial is summarized in Tables 1. Although this association is well recognized, the mechanisms underlying these complications remain incompletely understood. Preclinical and translational studies have proposed several plausible etiologies, including endothelial dysfunction, platelet activation, inflammatory signaling, and accelerated atherosclerosis. However, these findings have not been definitively validated in humans, and the pathophysiology remains uncertain [4, 5, 8]. Therefore, the vascular abnormalities observed in this patient should be interpreted as temporally associated with ponatinib exposure rather than conclusively caused by the medication.

**Table 1:** Reported incidence of major vascular adverse events associated with ponatinib in the Phase II PACE trial (1, 4, and 5).

Side effect	Reported incidence
Arterial occlusive event	17%–21%
Hypertension	25%
Heart failure	9%
Peripheral arterial occlusive disease	11%

The present case is notable because the patient experienced both myocardial infarction requiring coronary artery bypass grafting and symptomatic intracranial arterial disease requiring superficial temporal artery to middle cerebral artery bypass during the course of ponatinib therapy. Although these events raise suspicion for ponatinib‐associated vasculopathy, alternative explanations including the patient′s underlying cardiovascular risk factors of hypertension, diabetes mellitus, and hyperlipidemia may also have contributed. The relatively well‐controlled nature of these comorbidities and the temporal sequence of events support the consideration of a drug‐associated process but cannot fully establish causality.

To our knowledge, we identified no previously published reports describing sequential coronary and cerebral surgical revascularization in a patient receiving ponatinib. Although a single case cannot determine causality, it expands the spectrum of clinically significant vascular events that may occur during ponatinib therapy and highlights the importance of close monitoring in at risk patients. This observation should therefore be considered hypothesis generating rather than evidence of a unique toxic effect. Additional pharmacovigilance studies and larger observational cohorts are needed to better define the relationship between ponatinib exposure and multifocal arterial disease.

## 4. Conclusion

This case describes sequential coronary and cerebral revascularization procedures occurring in temporal association with ponatinib therapy in a patient with PH + ALL. Although a causal relationship cannot be inferred, the chronology of events raises concern for a possible ponatinib‐associated vasculopathy in the context of known cardiovascular risk factors. Clinicians prescribing ponatinib should remain vigilant for vascular complications, particularly in patients with preexisting cardiovascular comorbidities, and should engage in shared decision‐making regarding the balance between therapeutic benefit and potential toxicity. Reporting similar cases may improve understanding of the spectrum of vascular events associated with ponatinib and help inform risk stratification and management strategies.

## Funding

No funding was received for this manuscript.

## Consent

Signed informed consent was obtained from the patient and can be provided

## Conflicts of Interest

The authors declare no conflicts of interest.

## Data Availability

Data sharing is not applicable to this article as no datasets were generated or analyzed during the current study.

## References

[1] Januzzi J. L. , Garasic J. M. , Kasner S. E. , McDonald V. , Petrie M. C. , Seltzer J. , Mauro M. , Croce K. , Berman E. , Deininger M. , Hochhaus A. , Pinilla-Ibarz J. , Nicolini F. , Kim D. W. , DeAngelo D. J. , Kantarjian H. , Xu J. , Hall T. , Srivastava S. , Naranjo D. , and Cortes J. , Retrospective Analysis of Arterial Occlusive Events in the PACE Trial by an Independent Adjudication Committee, Journal of Hematology & Oncology. (2022) 15, no. 1, 10.1186/s13045-021-01221-z, 34991679. PMC8734305 34991679

[2] Network N. C. C. , Acute Lymphoblastic Leukemia, Version 1.2026, NCCN Clinical Practice Guidelines in Oncology 2026, https://www.nccn.org/professionals/physician_gls/pdf/all.pdf.

[3] Saleh K. , Fernandez A. , and Pasquier F. , Treatment of Philadelphia Chromosome-Positive Acute Lymphoblastic Leukemia in Adults, Cancers. (2022) 14, no. 7, 10.3390/cancers14071805, 35406576. PMC8997772 35406576

[4] Saussele S. , Haverkamp W. , Lang F. , Koschmieder S. , Kiani A. , Jentsch-Ullrich K. , Stegelmann F. , Pfeifer H. , la Rosée P. , Goekbuget N. , Rieger C. , Waller C. F. , Franke G. N. , le Coutre P. , Kirchmair R. , and Junghanss C. , Ponatinib in the Treatment of Chronic Myeloid Leukemia and Philadelphia Chromosome-Positive Acute Leukemia: Recommendations of a German Expert Consensus Panel With Focus on Cardiovascular Management, Acta haematologica. (2020) 143, no. 3, 217–231, 10.1159/000501927, 31590170. 31590170 PMC7384349

[5] Singh A. P. , Umbarkar P. , Tousif S. , and Lal H. , Cardiotoxicity of the BCR-ABL1 Tyrosine Kinase Inhibitors: Emphasis on Ponatinib, International Journal of Cardiology. (2020) 316, 214–221, 10.1016/j.ijcard.2020.05.077, 32470534. 32470534 PMC8095092

[6] Chan O. , Talati C. , Isenalumhe L. , Shams S. , Nodzon L. , Fradley M. , Sweet K. , and Pinilla-Ibarz J. , Side-Effects Profile and Outcomes of Ponatinib in the Treatment of Chronic Myeloid Leukemia, Blood Advances. (2020) 4, no. 3, 530–538, 10.1182/bloodadvances.2019000268, 32045474. 32045474 PMC7013263

[7] Jain P. , Kantarjian H. , Boddu P. C. , Nogueras-Gonzalez G. M. , Verstovsek S. , Garcia-Manero G. , Borthakur G. , Sasaki K. , Kadia T. M. , Sam P. , and Ahaneku H. , Analysis of Cardiovascular and Arteriothrombotic Adverse Events in Chronic-Phase CML Patients After Frontline TKIs, Blood Advances. (2019) 3, no. 6, 851–861, 10.1182/bloodadvances.2018025874, 30885996. 30885996 PMC6436011

[8] Zeng P. and Schmaier A. , Ponatinib and Other CML Tyrosine Kinase Inhibitors in Thrombosis, International Journal of Molecular Sciences. (2020) 21, no. 18, 10.3390/ijms21186556, 32911643. PMC7555546 32911643

